# Are men who have sex with men at higher risk for HIV in Latin America more aware of PrEP?

**DOI:** 10.1371/journal.pone.0255557

**Published:** 2021-08-13

**Authors:** Ryan D. Assaf, Kelika A. Konda, Thiago S. Torres, E. Hamid Vega-Ramirez, Oliver A. Elorreaga, Dulce Diaz-Sosa, Steven D. Diaz, Cristina Pimenta, Rebeca Robles, Maria Elena Medina-Mora, Beatriz Grinsztejn, Carlos Caceres, Valdilea G. Veloso

**Affiliations:** 1 University of California Los Angeles, Los Angeles, CA, United States of America; 2 Centro de Investigación Interdisciplinaria en Sexualidad Sida y Sociedad, UPCH, Lima, Peru; 3 Instituto Nacional de Infectologia Evandro Chagas, Fundação Oswaldo Cruz (INI-Fiocruz), Rio de Janeiro, Brazil; 4 Instituto Nacional de Psiquiatria Ramon de la Fuente Muñiz, Mexico City, Mexico; 5 United Nations Population Fund (UNFPA Mexico) of Mexico City, Mexico City, Mexico; 6 Ministério da Saúde Brazil, Brasília, Brazil; The Chinese University of Hong Kong, CHINA

## Abstract

**Introduction:**

PrEP awareness in Latin America has been poorly characterized, with studies in Brazil, Mexico, and Peru highlighting awareness of 65% among gay, bisexual and other men who have sex with men (MSM). We assessed the association between higher risk of HIV infection, indicative of PrEP eligibility, and PrEP awareness among MSM from these countries.

**Methods:**

This was a secondary analysis of a web-based survey advertised on social media platforms from March-June 2018 in Brazil, Mexico and Peru. Eligible individuals were cisgender MSM, ≥18 years old, HIV negative or of unknown status, who lived in these countries, and provided informed consent. Higher risk of HIV infection was defined as having 10 or more points in the HIV Risk Index for MSM (HIRI-MSM). We used multivariable Poisson regression models to calculate adjusted prevalence ratios (aPR) testing the association between higher risk for HIV and PrEP awareness.

**Results:**

After exclusions, 19,457 MSM were included in this analysis. In Brazil, 53.8% were classified as higher risk for HIV, 51.9% in Mexico, and 54.2% in Peru. Higher risk for HIV was minimally associated with PrEP awareness among those in Brazil (aPR 1.04, 95% CI 1.01, 1.06), but no such association was observed in Mexico or Peru. Having more than a high school education, high income, daily use of geosocial networking (GSN) applications, and substance use were associated with PrEP awareness.

**Conclusion:**

Higher risk of HIV infection was associated with increased PrEP awareness in Brazil. However, this association was weak indicating that PrEP awareness could be strengthened with further prevention efforts. In the remaining countries, results were non-conclusive between risk and awareness. Interventions to increase PrEP awareness are paramount to increase PrEP willingness and uptake and in turn prevent new HIV infections. Social media platforms could play an important role to achieve this goal.

## Introduction

Human immunodeficiency virus (HIV) remains a global pandemic. In 2018, there were 37.9 million people living with HIV and 770,000 HIV-related deaths worldwide, with 2.2 million cases of HIV and 100,000 new infections in Latin America [1, 2]. Brazil, Mexico, and Peru had an estimated 900,000, 230,000 and 79,000 HIV cases respectively in 2018 [3–5]. Gay, bisexual, and other men who have sex with men (MSM) are disproportionally impacted by HIV in Latin America [1, 6–13] and are 33.3 times more likely to be infected with HIV compared to the general population [14]. This risk for HIV may be an underestimate as MSM in Latin America continually face social stigmatization and may not seek HIV testing or treatment [14, 15].

Although the global burden of HIV persists, oral pre-exposure prophylaxis (PrEP) for HIV with tenofovir and emtricitabine (TDF/FTC) is a safe and effective tool for biomedical prevention of HIV [16–21]. For MSM even non-perfect adherence provides protection against HIV by more than 90% [22]. PrEP use has been recommended by the World Health Organization (WHO) in 2014 as an additional tool to prevent HIV among MSM and was revised in 2015 to include any high-risk population [15, 23, 24]. Brazil became the first country in Latin America to include freely available PrEP for high risk populations including MSM and transgender women in 2018 (TGW) [25]. A large scale PrEP implementation project in Brazil, Mexico and Peru started in 2018 and is currently in progress (www.imprep.org) [26].

A previous PrEP demonstration project conducted in Brazil from 2014 to 2015 among MSM and TGW, showed that prior PrEP awareness was associated with PrEP uptake (adjusted odds ratio [aOR] 2.19, 95% confidence interval [CI] 1.52, 3.16); those who were eligible for the study, but choose not to enroll were younger and less educated (all p-values<0.05). This study concluded that for PrEP to be effective, efforts to raise PrEP awareness among younger and less educated MSM and TGW were needed [27]. Factors previously shown to be associated with PrEP awareness in Brazil included older age, more years of education, higher income, marijuana use, and high risk sexual behavior, measured by having an HIV positive partner and having more than five recent sex partners [28–30]. In Peru, PrEP awareness was assessed in focus groups with Peruvian MSM, TGW, and female sex workers; respondents in all three groups indicated little to no knowledge or awareness of PrEP. This study was conducted prior to PrEP being proven efficacious and WHO’s 2014 PrEP recommendation [31]. PrEP awareness in Latin America was assessed in 2012 among residents in Brazil, Mexico, and Colombia; survey results showed overall PrEP awareness of 10.4% among MSM mostly of high education and of middle to high income [32].

We have previously shown that PrEP awareness was 64.9% in 2018 among MSM from Brazil, Mexico and Peru, being higher in Brazil (68.8%), followed by Mexico (64.1%), and Peru (46.6%). Almost half of respondents were classified as higher risk for HIV and willingness to use PrEP was strongly associated with PrEP awareness in Brazil (aOR 1.66; 95% CI 1.52–1.81), Mexico (aOR 2.05; 95%CI 1.79–2.34) and Peru (aOR 1.84; 95%CI 1.51–2.26) [33]. Although Brazil has some data available [27–30], most of the countries in Latin America, including Mexico and Peru, have limited information about factors associated with PrEP awareness. In our prior analysis, the focus was on factors associated with willingness to use PrEP. The objective of this secondary analysis is to assess the association between MSM at higher risk for HIV and awareness of PrEP among those in Brazil, Mexico, and Peru with the goal of providing information to identify gaps in awareness, which could help public health educational campaigns target those most in need of PrEP. Additionally, we aim to assess which other factors are associated with awareness of PrEP in these populations.

## Materials and methods

### Study design and population

This is a secondary analysis of data collected via a web-based survey as part of the formative research for the ImPrEP project in Brazil, Mexico, and Peru. The methods of this study and detailed description of study variables have been previously described but will be briefly mentioned here [33]. Respondents were recruited through advertisements on Facebook^©^, Grindr, ^©^ and Hornet^©^ from March to June 2018. Additionally, respondents were able to share the survey link with others if desired. Respondents were eligible for the study if they met the following criteria: (a) 18 or older in age; (b) cisgender men; (c) self-reported HIV negative; and (d) resided in Brazil, Mexico, or Peru. The informed consent stated clearly that this survey was targeting cisgender men and invited transgender individuals to answer a parallel survey; however, that survey did not have sufficient response to include the information in this analysis. All participants that had complete data were included for this study. More detail of exclusion/inclusion criteria have been explained previously [30]. The study protocol was reviewed and approved by the Institutional Review Boards at the participating institutions: Brazil, INI Evandro Chagas-FIOCRUZ institutional review board (#82021918.0.0000.5262 at ‘‘Plataforma Brasil”); Mexico, the research ethics committee of the National Institute of Psychiatry (#CEI/C/038/2018); Peru, UPCH Ethical Committee for Research with Human Subjects approval #101460. All respondents provided digital informed consent prior to initiating the survey.

### Data collection

Data collection consisted of a 10 to 15-minute anonymous web-based survey; those from Brazil responded to the survey in Portuguese via SurveyGizmo^®^ and those from Mexico and Peru responded in Spanish via SurveyMonkey^®^. No personally identifying information was collected. Participants answered questions on demographics, sexual behaviors, HIV testing and STI status, as well as awareness, willingness to use, and past or current use of PrEP.

### Variable definitions and categorization

The primary outcome was the respondent’s awareness of PrEP. For comparison, participants were dichotomized into those who indicated they were aware of PrEP and those who indicated they were not aware of PrEP. PrEP awareness was assessed with the following questions: “Have you ever heard of PrEP?”. Upon answering this question, respondents were presented with a brief explanation of PrEP and had the ability to go back and revise their answer if desired.

Our exposure of interest was higher risk for HIV acquisition defined using the HIV Incidence Risk Index for MSM score (HIRI-MSM) and the suggested [29, 30] cut-point of 10 for increased risk of HIV infection [34]. The HIRI-MSM score is dependent on the respondent’s age, the number of male sex partners in the last 6 months, the number of times the respondent had receptive anal sex with another male in the last 6 months, the number of HIV positive male sex partners, the number of times the participant had insertive anal sex with another male in the last 6 months, methamphetamine use in the last 6 months, and use of poppers in the last 6 months. Cocaine was used in addition to methamphetamine as methamphetamine use was virtually non-existent in our study population [29, 30, 35–40]. The HIRI-MSM score has been previously used by the Unite States Center for Disease Control and Prevention (CDC) and New York State Department of Health as a tool to identify eligible candidates for PrEP [30, 41]. Previous studies have identified that a respondent’s risk perception for HIV tends to be less than their calculated HIRI-MSM score [41, 42]. Thus, the HIRI-MSM score is an impartial calculator dependent on respondent’s behavior, instead of being based on their risk perception.

Other variables of interest included demographics, substance use, use of geosocial networking (GSN) applications (apps) to find sex partners, and binge drinking which have been previously defined [33]. Briefly age was based on self-report. Income was based on multiples of the minimum wage in each country. Education was assessed by five categories (less than high school, high school, did not complete undergraduate, completed undergraduate, and postgraduate degree). Race was included in this analysis as a sociopolitical framework to be a proxy control for social, economic, and structural disparities that exists between groups [43, 44]. Race was collected differently in Mexico compared to Brazil and Peru. For Mexico, race was dichotomized (White/other and Indigenous), while being stratified into four categories in Brazil and Peru (White, Black, Brown or “Pardo”, and other). The use of geosocial networking (GSN) applications (apps) to find sex partners was based on number of days individuals reported this use. Binge drinking was based on number of times an individual had more than five drinks in a 2-hour period. Substance use was measured for a variety of drugs [45]. Use of cocaine in any form was included in the HIRI-MSM score. In this analysis substance use was evaluated for erectile dysfunction drugs and/or marijuana, as recreational use of these drugs during sex have been previously reported in Latin America. Other illicit drugs were not included in this analysis due to the low frequency (<1% reported use of hallucinogens, GHB, ecstasy, heroin, ketamine, and inhalants) in the last 6 months [37, 46].

### Variable selection and statistical analyses

Demographic and variable distributions among the populations in each country were analyzed using frequencies, median, and interquartile ranges (IQR). Bivariate analyses used Pearson’s chi-squared tests to compare participant characteristics and behaviors with PrEP awareness. Bivariate and multivariable modeling was performed using generalized linear modeling (GLM) with Poisson regression and robust adjustment. Poisson regression with robust adjustment was used to obtain efficient results closest to the Mantel-Hansel prevalence ratio [47]. Results are presented as prevalence ratios (PR), adjusted prevalence ratios (aPR), and 95% confidence intervals (CI). Covariates were included in the model based on *a priori* knowledge and existing literature on factors associated with PrEP awareness. Directed acyclic graphs (DAGs) were used to identify confounders, mediators, and colliders [48]. Age, education, race, income, use of GSN apps, substance use, and binge drinking were considered potential confounders. HIV testing has been previously shown to have a strong association with PrEP awareness [28, 30]. However, for this specific analysis HIV testing was believed to be a mediator and was purposefully not included in the analysis as it could reduce the effect of the interest. A mediation analysis of HIV testing was conducted to assess the mediator effects of HIV testing between HIRI-MSM risk and PrEP awareness [49, 50]. However, the portion of the effect due to HIV testing as a mediator was not significant for any country, therefore this analysis is not shown.

Separate multivariable models by country were constructed for Brazil, Mexico, and Peru. For the multivariable analysis, given the limited number of individuals reporting less than high school education, especially in Peru, less than high school and high school were collapsed into one category. Finally, missing data for the exposure of interest (HIRI-MSM risk) was 2.52%, 1.33%, and 0.70% for Brazil, Mexico, and Peru, respectively. Missing data for the outcome of interest (PrEP awareness) was 0.37%, 0.22%, and 0.28% for Brazil, Mexico, and Peru, respectively. Missing data primarily occurred due to missingness on income, a covariate for confounder control. With minimal missing data on exposure or outcome, we did not believe missingness would negatively impact our analysis. All analyses were conducted using STATA v.14 (STATA, College Station Texas, US).

## Results

Of the 43,687 respondents who started the survey, 19,457 met the inclusion criteria described above and completed the survey. Of those who initiated the survey 8790 (20.7%) were ineligible, while among eligible respondents 44.2% did not complete the survey. Non-completers were: younger, had lower education levels, were less likely to be white, and were of lower SES. Respondents in Brazil, Mexico, and Peru were reflective of each countries’ regions where the HIV epidemic is most prominent. The median age among study participants was 28 years old (IQR 24–34). Most participants were from Brazil (58.4%), followed by Mexico (30.5%) and Peru (11.1%). A large portion of respondents had a postsecondary degree (59.9%) and only a few did not complete high school (1.5%). Most respondents identified having middle (43.1%) or low (39.6%) income.

Overall, the risk of participants based on the HIRI-MSM indicated that 53.3% of the population would be at higher risk of HIV, but only 24.7% perceived themselves to be at moderate risk and 9.6% at higher risk of HIV. In Brazil, 53.8% of respondents were classified on the HIRI-MSM as being at higher risk for HIV, 51.9% in Mexico, and 54.2% in Peru.

Those from Brazil and Mexico who were 31–35 years of age reported the highest awareness of PrEP (75.4% and 57.8% respectively). However, in Peru, those who were 36 years or older had the highest awareness (57.8%). Respondents who reported their race as White in Brazil and Peru reported highest awareness of PrEP (71.6% and 52.2%, respectively). Among the three countries, those who reported higher income also reported higher awareness of PrEP in Brazil (80.6%), Mexico (79.3%), and Peru (70.2%). Respondents who reported daily use of GSN apps reported higher awareness of PrEP in Brazil (70.5%), Mexico (66.7%), and Peru (52.5%). Finally, respondents who reported use of both erectile dysfunction drugs and marijuana reported higher awareness of PrEP: 87.1% in Brazil, 85.1% in Mexico, and 75.9% in Peru (Table 1).

**Table 1:** Frequency distributions of PrEP awareness by demographics and behaviors compared to those not aware of PrEP: Brazil, Mexico, Peru (N = 19,457) April, 2018.

	Brazil (N = 7,794/11,325)			Mexico (N = 3,796/5,921)			Peru (N = 1,002/2,150)		
	n/N*	Aw%**	P value	n/N*	Aw%**	P value	n/N*	Aw%**	P value
PrEP Awareness (N)	7,794	68.8	--	3,796	64.1	--	1,002	46.6	--
***Sociodemographic Characteristics***									
Age Category			<0.001			<0.001			<0.001
36+	1,975/7,794	71.3		776/3,796	65.9		171/1,002	57.8	
31–35	1,495/7,794	75.4		679/3,796	70.3		134/1,002	52.6	
25–30	2,381/7,794	70.7		1,365/3,796	67.7		370/1,002	51.9	
18–24	1,942/7,794	60.7		976/3,796	55.4		327/1,002	36.9	
Race (Brazil & Peru)			<0.001			--			0.008
White	4,288/7,794	71.6		--	--		212/1,002	52.2	
Black	996/7,794	66.9		--	--		11/1,002	26.8	
^c^Mix	2,290/7,794	65.1		--	--		719/1,002	46.1	
^d^Other	110/7,794	65.9		--	--		30/1,002	46.2	
Race (Mexico Only)			--			0.002			--
White/Other	--	--		3,717/3,796	64.4		--	--	
Indigenous	--	--		56/3,796	50.0		--	--	
Education			<0.001			<0.001			<0.001
Postgraduate	2,259/7,794	79.6		706/3,796	78.5		151/1,002	63.7	
Completed undergraduate	2,939/7,794	72.9		1,830/3,796	69.6		487/1,002	53.8	
Did not complete undergraduate	478/7,794	62.8		1,219/3,796	54.5		185/1,002	35.7	
Completed high school	1,957/7,794	58.3		20/3,796	22.0		165/1,002	36.6	
Less than high school	102/7,794	42.9		11/3,796	27.5		1/1,002	12.5	
Income			<0.001			<0.001			<0.001
High	1,232/7,794	80.6		1,124/3,796	79.3		191/1,002	70.2	
Middle	3,473/7,794	74.1		1,544/3,796	64.5		448/1,002	47.4	
Low	3,089/7,794	60.5		821/3,796	53.3		259/1,002	36.6	
***Behaviors and HIV Testing***									
GSN Apps for Sex			<0.001			<0.001			<0.001
Daily	4,315/7,794	70.5		1,441/3,796	66.7		372/1,002	52.5	
Sometimes	2,983/7,794	68.3		2,118/3,796	63.6		491/1,002	47.8	
Never	496/7,794	59.8		237/3,796	55.4		139/1,002	33.6	
^a^Binge Drinking			<0.001			0.005			0.3
Yes	5,422/7,794	69.9		2,670/3,796	65.3		694/1,002	45.9	
No	2,372/7,794	66.4		1,105/3,796	61.5		293/1,002	48.3	
^a^Substance use			<0.001			<0.001			<0.001
Erectile dysfunction use only	505/7,794	76.8		280/3,796	74.3		34/1,002	55.7	
Marijuana use only	1,855/7,794	74.1		820/3,796	71.9		246/1,002	55.9	
Marijuana and erectile dysfunction use	344/7,794	87.1		194/3,796	85.1		22/1,002	75.9	
No use	5,090/7,794	65.5		2,502/3,796	59.9		700/1,002	43.2	
***Sexual Behaviors and Risk***									
^b^Perceived HIV Risk			0.7			0.008			0.7
High	690/7,794	70.0		426/3,796	67.2		106/1,002	44.5	
Middle	1,580/7,794	68.9		1,215/3,796	66.1		310/1,002	47.6	
Low	5,524/7,794	68.7		2,155/3,796	62.5		586/1,002	46.5	
^a^MSM Risk Index (HIRI-MSM)			<0.001			0.001			0.5
Higher	4,237/7,794	71.2		2,005/3,796	66.0		547/1,002	47.2	
Lower	3,381/7,794	66.4		1,741/3,796	62.1		446/1,002	45.7	

Abbreviations. MSM = Men who have Sex with Men; PrEP = Pre-Exposure Prophylaxis; HIV = Human Immunodeficiency Virus; GSN = Gay Social Networking; HIRI-MSM = HIV Incidence Risk Index for Men who have Sex with Men

*May not add to total because of missing data

** Aw% = Percentages are indicators of those aware to those not aware in that category

^a^Variables asked in the previous 6 months (binge drinking, substance use, MSM risk index)

^b^Variables asked in the previous 12 months (perceived risk)

^c^Mix race included respondents who stated their race as “mestizo”

^d^Other race included respondents who stated their race was other

Risk for HIV, age, race, education, income, use of GSN apps, binge drinking, and substance use were associated with PrEP awareness in the bivariate models among those in Brazil and those in Mexico (p-values<0.05) (Tables 2 and 3). In Peru, age, race, education, income, use of GSN apps, and substance use were associated with PrEP awareness in the bivariate models (p-value <0.05) (Table 4).

**Table 2:** PrEP awareness among MSM in Brazil, crude and adjusted prevalence ratios (n = 11,325) April, 2018.

	Crude PR		Adjusted Model: Brazil (n = 10,780*)	
	Crude PR	95% CI	Adjusted PR	95% CI
^a^MSM Risk Index (HIRI-MSM)				
Low	1.00	Ref	1.00	Ref
High	1.07	(1.05, 1.10)	**1.04**	**(1.01, 1.06)**
Age, years				
36+	1.17	(1.13, 1.22)	1.01	(0.97, 1.05)
31–35	1.24	(1.20, 1.29)	**1.07**	**(1.03, 1.12)**
25–30	1.17	(1.13, 1.21)	**1.04**	**(1.01, 1.08)**
18–24	1.00	Ref	1.00	Ref
Education				
Postgraduate	1.39	(1.34, 1.44)	**1.26**	**(1.22, 1.32)**
Completed undergraduate	1.27	(1.23, 1.32)	**1.20**	**(1.16, 1.24)**
Completing undergraduate	1.10	(1.03, 1.17)	**1.07**	**(1.01, 1.14)**
High school or less	1.00	Ref	1.00	Ref
Race				
White	1.00	Ref	1.00	Ref
Black	0.93	(0.90, 0.97)	1.01	(0.97, 1.05)
Mix	0.91	(0.88, 0.94)	0.96	(0.93, 0.99)
Other	0.92	(0.82, 1.03)	0.95	(0.86, 1.06)
Income				
High	1.09	(1.06, 1.12)	**1.06**	**(1.03, 1.09)**
Middle	1.00	Ref	1.00	Ref
Low	0.82	(0.79, 0.84)	**0.89**	**(0.86, 0.92)**
GSN Apps for Sex				
Never	0.88	(0.83, 0.93)	0.92	(0.87, 0.97)
Sometimes	1.00	Ref	1.00	Ref
Daily	1.03	(1.01, 1.06)	**1.03**	**(1.01, 1.06)**
Binge Drinking				
No	1.00	Ref	1.00	Ref
Yes	1.05	(1.02, 1.08)	1.02	(0.99, 1.05)
Substance Use				
No	1.00	Ref	1.00	Ref
Erectile dysfunction use only	1.17	(1.12, 1.23)	**1.08**	**(1.03, 1.13)**
Marijuana use only	1.13	(1.10, 1.16)	**1.12**	**(1.09, 1.15)**
Marijuana and erectile dysfunction use	1.33	(1.28, 1.39)	**1.21**	**(1.16, 1.26)**

Abbreviations. PrEP = Pre-Exposure Prophylaxis; MSM = Men who have Sex with Men; HIV = Human Immunodeficiency Virus; PR = Prevalence ratios; HIRI-MSM = HIV Incidence Risk Index for Men who have Sex with Men; GSN = Gay Social Networking

* May not add to total because of missing data

^a^ Adjusted for: age, education, race, income, GSN apps for sex, binge drinking, substance use

**Table 3:** PrEP awareness among MSM in Mexico, crude and adjusted prevalence ratios (n = 5,921) April, 2018.

	Crude PR		Adjusted Model: Mexico (n = 5,220*)	
	Crude PR	95% CI	Adjusted PR	95% CI
^a^MSM Risk Index (HIRI-MSM)				
Low	1.00	Ref	1.00	Ref
High	1.06	(1.02, 1.11)	1.02	(0.98, 1.06)
Age, years				
36+	1.19	(1.12, 1.26)	0.95	(0.88, 1.02)
31–35	1.27	(1.20, 1.35)	1.04	(0.97, 1.11)
25–30	1.22	(1.16, 1.29)	1.05	(0.99, 1.11)
18–24	1.00	Ref	1.00	Ref
Education				
Postgraduate	3.32	(2.44, 4.52)	**2.86**	**(2.06, 3.97)**
Completed undergraduate	2.94	(2.16, 4.00)	**2.64**	**(1.91, 3.67)**
Completing undergraduate	2.30	(1.69, 3.14)	**2.23**	**(1.61, 3.10)**
High school or less	1.00	Ref	1.00	Ref
Race (Mexico only)				
White/other	1.00	Ref	1	Ref
Indigenous	0.78	(0.64, 0.94)	0.87	(0.73, 1.04)
Income				
High	1.23	(1.18, 1.28)	**1.16**	**(1.11, 1.21)**
Middle	1.00	Ref	1.00	Ref
Low	0.83	(0.78, 0.87)	**0.91**	**(0.86, 0.96)**
GSN Apps for Sex				
Never	0.87	(0.80, 0.95)	**0.91**	**(0.83, 0.99)**
Sometimes	1.00	Ref	1.00	Ref
Daily	1.05	(1.01, 1.09)	1.02	(0.98, 1.06)
Binge Drinking				
No	1.00	Ref	1.00	Ref
Yes	1.06	(1.02, 1.11)	0.99	(0.95, 1.04)
Substance Use				
No use	1.00	Ref	1.00	Ref
Erectile dysfunction use only	1.24	(1.16, 1.32)	**1.16**	**(1.08, 1.24)**
Marijuana use only	1.20	(1.15, 1.25)	**1.17**	**(1.12, 1.23)**
Marijuana and erectile dysfunction use	1.42	(1.34, 1.51)	**1.33**	**(1.24, 1.42)**

Abbreviations. PrEP = Pre-Exposure Prophylaxis; MSM = Men who have Sex with Men; HIV = Human Immunodeficiency Virus; PR = Prevalence ratios; HIRI-MSM = HIV Incidence Risk Index for Men who have Sex with Men; GSN = Gay Social Networking

May not add to total because of missing data

^a^ Adjusted for: age, education, race, income, GSN apps for sex, binge drinking, substance use

**Table 4:** PrEP awareness among MSM in Peru, crude and adjusted prevalence ratios (n = 2,150) April, 2018.

	Crude PR		Adjusted Model: Peru (n = 1,812*)	
	Crude PR	95% CI	Adjusted PR	95% CI
^a^MSM Risk Index (HIRI-MSM)				
Low	1.00	Ref	1.00	Ref
High	1.03	(0.94, 1.13)	0.97	(0.88, 1.07)
Age, years				
36+	1.57	(1.37, 1.78)	1.13	(0.95, 1.33)
31–35	1.42	(1.23, 1.65)	1.07	(0.91, 1.27)
25–30	1.41	(1.26, 1.57)	**1.14**	**(1.00, 1.30)**
18–24	1.00	Ref	1.00	Ref
Education				
Postgraduate	1.76	(1.51, 2.06)	1.13	(0.93, 1.38)
Completed undergraduate	1.49	(1.30, 1.70)	1.17	(0.99, 1.38)
Completing undergraduate	0.99	(0.84, 1.17)	0.85	(0.71, 1.03)
High school or less	1.00	Ref	1.00	Ref
Race				
White	1.00	Ref	1.00	Ref
Black	0.51	(0.31, 0.86)	0.62	(0.38, 1.03)
Mix	0.88	(0.79, 0.98)	0.90	(0.80, 1.00)
Other	0.88	(0.67, 1.17)	0.85	(0.62, 1.18)
Income				
High	1.48	(1.34, 1.64)	**1.33**	**(1.18, 1.50)**
Middle	1.00	Ref	1.00	Ref
Low	0.77	(0.69, 0.87)	**0.85**	**(0.74, 0.97)**
GSN Apps for Sex				
Never	0.70	(0.61, 0.82)	0.74	(0.63, 0.87)
Sometimes	1.00	Ref	1.00	Ref
Daily	1.10	(1.00, 1.21)	1.09	(0.99, 1.20)
Binge Drinking				
No	1.00	Ref	1.00	Ref
Yes	0.95	(0.86, 1.05)	**0.87**	**(0.79, 0.97)**
Substance Use				
No use	1.00	Ref	1.00	Ref
Erectile dysfunction use only	1.29	(1.02, 1.62)	0.99	(0.77, 1.28)
Marijuana use only	1.29	(1.17, 1.43)	**1.35**	**(1.21, 1.50)**
Marijuana and erectile dysfunction use	1.76	(1.42, 2.17)	**1.58**	**(1.29, 1.94)**

Abbreviations. PrEP = Pre-Exposure Prophylaxis; MSM = Men who have Sex with Men; HIV = Human Immunodeficiency Virus; PR = Prevalence ratios; HIRI-MSM = HIV Incidence Risk Index for Men who have Sex with Men; GSN = Gay Social Networking

May not add to total because of missing data

^a^ Adjusted for: age, education, race, income, GSN apps for sex, binge drinking, substance use

In the multivariable models, higher risk for HIV was minimally associated with PrEP awareness in Brazil compared to those who were lower risk for HIV, aPR 1.04 (95% CI 1.01, 1.06) (Table 2). However, in Mexico and Peru, higher risk for HIV was not associated with PrEP awareness, aPR 1.02 (95% CI 0.98, 1.06) and aPR 0.97 (95% CI 0.88, 1.07), respectively (Tables 3 and 4).

In Brazil and Peru, being 25–30 years of age was associated with an increased aPR of PrEP awareness compared to those who were 18–24 years of age. Additionally, in Brazil, being 31–35 years of age was positively associated with PrEP awareness compared to those who were 18–24 years of age (Tables 2 and 4). In Brazil and Mexico, completing undergraduate, completed undergraduate, and having a postgraduate degree were all associated with increased PR of PrEP awareness compared to those with a high school degree or less (Tables 2 and 3). In all three countries, having a higher income was positively associated with PrEP awareness, while low income was negatively associated with PrEP awareness compared to middle income (Tables 2–4). Daily use of GSN apps to find sex partners among those from Brazil was positively associated with PrEP awareness compared to those who sometimes used GSN apps (Table 2). In Mexico and Peru, never use of GSN apps to find sex partners was negatively associated with PrEP awareness compared to those who sometimes used GSN apps (Tables 3 and 4). In Peru, binge drinking was negatively associated with PrEP awareness compared to those who did not binge drink (Table 4). Finally, in all three countries, those who used marijuana only and those who used both marijuana and erectile dysfunction drugs were positively associated with PrEP awareness compared to those who did not use either. In addition, those in Brazil and Mexico who reported erectile dysfunction drug use only were associated with an increased PR of PrEP awareness compared to those who did not use either marijuana or erectile dysfunction drugs (Tables 2–4).

## Discussion

Our study highlights the association with higher risk for HIV among MSM and PrEP awareness prior to PrEP being widely available in Brazil. Those at higher risk for HIV were more aware of PrEP in Brazil, although the point estimate for this association was small (aPR 1.04). In Mexico and Peru, higher risk for HIV was not associated with PrEP awareness. PrEP awareness among MSM at higher risk for HIV is often the first step needed to increase PrEP demand and PrEP uptake, contributing to the prevention of new HIV cases.

It is likely that the association of higher risk for HIV and PrEP awareness between these three countries reflects the duration of PrEP availability and accessibility in each. Brazil has had PrEP for the longest time and has made it available at the national level, whereas Mexico and Peru have only recently implemented PrEP in a few sites [25, 26]. These differences may explain the greater overall percentage of awareness in Brazil in comparison to Mexico and Peru. This analysis demonstrates that information about PrEP needs to be further disseminated to increase levels of awareness. As PrEP implementation in these three countries continues and increases, awareness of PrEP will also increase. As seen in previous studies, PrEP awareness is associated with PrEP uptake and is strongly associated with willingness to use PrEP [27–29, 33]. Therefore, identifying factors related to PrEP awareness and efforts on increasing awareness is an important first step in targeting PrEP scale-up.

This study contributes to important findings about variables that are associated with PrEP awareness. PrEP awareness has been previously shown to be associated with older age, higher education, and higher income among those from Brazil [28–30]). These findings held true in our study. In Peru, PrEP awareness was associated with older age and in Mexico higher education was positively associated with PrEP awareness. In all three countries, higher income was positively associated with PrEP awareness, while lower income was negatively associated with PrEP awareness. These associations highlight the link between higher education and higher income and access to information about PrEP. This is of concern because individuals with lower education and lower income are also at high risk of HIV and need information on PrEP. Individuals with lower income may have less access to health services compared to those who are of higher income [32]. Future interventions should focus on reaching these groups [27, 51].

Other variables associated with PrEP awareness in our study population included use of GSN apps and substance use. Daily use of GSN apps to find sex partners was positively associated with PrEP awareness in Brazil. Agreeing with other literature, this finding shows that GSN applications may be used as a platform to spread awareness of PrEP and education [29, 52, 53]. Exposure to PrEP information in GSN applications may occur through advertisements or in users’ profiles where they indicate PrEP use [52, 54]. Further, never use of GSN applications showed a negative association with PrEP awareness in Mexico and Peru. Thus, individuals who never use GSN applications may be less exposed to PrEP information while those who use GSN applications daily are more exposed to information. Use of GSN applications may also be a proxy for social factors that would increase exposure to PrEP information [52]. Substance use (marijuana and/or erectile dysfunction drug use) was positively associated with PrEP awareness in all three countries. Use of these substances may reflect proxy associations with PrEP awareness due to social and behavioral factors other than just using the substances. As shown previously in Brazil, having more than 5 partners in the previous 6 months, having more friends with the same sexual orientation, and marijuana use is associated with PrEP awareness [30]. Recreational use of erectile dysfunction drugs can occur in junction with group sex and may be associated with having an increased number of sexual partners [46]. Thus, it is possible that one’s exposure to others (which may include friends/partners with the same sexual orientation) who may know about or use PrEP, increases their awareness of PrEP.

This study was made available to individuals on social media platforms such as Facebook^©^ and GSN apps such as Grindr^©^ and Hornet^©^. There was no monetary incent for survey participation. Over 40,000 participants started the survey and 19,457 were included in the analyses. Information bias, specifically social desirability bias, may be limited because the surveys were anonymous and filled out at the individuals’ will. No identifying information was collected; therefore, the effect of stigma and other social factors may be reduced. Finally, we were able to obtain large sample sizes from all three countries throughout the different regions of the countries. The distribution of higher risk individuals reflected the regions where HIV is most prevalent in these countries and where PrEP is currently being made available.

This study has limitations that must be acknowledged. First, there may be selection bias. Those who did not complete the survey were younger, had lower education levels, were less likely to be white, and were of lower SES. However, control for these variables may remove some of the effect of selection bias. Second, additional barriers related to PrEP awareness such as stigma and homonegativity were not collected in this survey and could not be evaluated. Third, all information collected on individuals was self-reported. We did not have access to medical records or other sources of data to crosscheck for misclassification. Recall bias may also impact the results as respondents were asked about exposures 3 months, 6 months, and 12 months prior to the survey. Fourth, this study may not be generalizable to all MSM population in the three countries due to convenience sampling. The study design aimed to expand coverage of the populations studied by making the survey available to participants in the three countries on multiple online forums and by allowing the survey to be disseminated to peers who may have not had access to these online sites. However, respondents needed access to a smartphone or computer and internet. Finally, there were some differences in how questions were posed between the countries. Specifically, income in Brazil was asked as family income, while income in Mexico and Peru was asked as individual income. Therefore, conclusions of income in Brazil are different than those for Mexico and Peru.

## Conclusion

This study showed MSM at higher risk for HIV may be slightly more aware of PrEP in Brazil. Results in Mexico and Peru were similar to one another as they were both inconclusive with regard to this association, which may reflect the lack of availability of PrEP in each of these countries at the time of the survey. PrEP can be a useful tool in ending the HIV epidemic in Latin America, but more work is needed to increase PrEP awareness among MSM who can benefit most of PrEP, such as MSM in higher risk, of younger age, lower income and lower education.

## Future direction

Increase PrEP awareness among MSM is central for PrEP implementation success. Despite economic and political instability in the three countries, PrEP access, implementation, and scale-up are currently underway. As PrEP accessibility increases in Mexico and Peru, it is predicted that awareness of PrEP will rise similar to that seen in Brazil, where PrEP has been implemented in the Public Health System available free of charge in all states. In Mexico, the current national HIV program has showed a commitment to follow and support ImPrEP project. Since this is the first PrEP project in Mexico, knowing the facilitators and barriers to increase awareness of PrEP among the potential users is crucial to understand for tailored strategies of implementation. In Peru, the Ministry of Health has signaled their interest in incorporating PrEP into the prevention strategy, having already included it for pregnant women in a sero-discordant partnership [55]. As new HIV prevention guidelines are redone, PrEP is expected to be included as part of the national strategy.

Future work should focus on reaching populations at higher risk for HIV and on identifying ways to increase PrEP awareness among those of lower income and lower education. Finally, the use of GSN apps can continue to be a useful platform to spread information about PrEP and its availability in these three countries.

## Supporting information

S1 Questions (XLSX)

## List of abbreviations

HIV: Human immunodeficiency virus
MSM: Men who have sex with men
PrEP: Pre-exposure prophylaxis
TDF/FTC: Tenofovir and emtricitabine
WHO: World Health Organization
TGW: Transgender women
aOR: Adjusted odds ratio
CI: Confidence interval
CDC: Centers for Disease Control and Prevention
GSN: Geosocial networking
IQR: Interquartile range
GLM: Generalized linear modeling
PR: Prevalence ratios
aPR: Adjusted prevalence ratios
DAGs: Directed acyclic graphs
